# Automated CRISPR/Cas9-based genome editing of human pluripotent stem cells using the StemCellFactory

**DOI:** 10.3389/fbioe.2024.1459273

**Published:** 2024-09-20

**Authors:** Bastian Nießing, Yannik Breitkreuz, Andreas Elanzew, Marcelo A. S. de Toledo, Peter Vajs, Marina Nolden, Frederik Erkens, Paul Wanek, Si Wah Christina Au Yeung, Simone Haupt, Niels König, Michael Peitz, Robert H. Schmitt, Martin Zenke, Oliver Brüstle

**Affiliations:** ^1^ Fraunhofer Institute for Production Technology (FHG), Aachen, Germany; ^2^ LIFE & BRAIN GmbH, Bonn, Germany; ^3^ Institute of Reconstructive Neurobiology, University of Bonn, Bonn, Germany; ^4^ Institute for Biomedical Engineering, Faculty of Medicine, RWTH Aachen University, Aachen, Germany; ^5^ Helmholtz-Institute for Biomedical Engineering, RWTH Aachen University, Aachen, Germany; ^6^ Department of Hematology, Oncology, Hemostaseology and Stem Cell Transplantation, University Hospital RWTH Aachen, Aachen, Germany; ^7^ Center for Integrated Oncology Aachen Bonn Cologne Düsseldorf (CIO ABCD), Aachen, Germany; ^8^ Cell Programming Core Facility, Faculty of Medicine, University of Bonn, Bonn, Germany; ^9^ Laboratory for Machine Tools and Production Engineering, RWTH Aachen University, Aachen, Germany

**Keywords:** genome editing, CRISPR/Cas9, automation, StemCellFactory, induced pluripotent stem cells, iPS cells

## Abstract

CRISPR/Cas9 genome editing is a rapidly advancing technology that has the potential to accelerate research and development in a variety of fields. However, manual genome editing processes suffer from limitations in scalability, efficiency, and standardization. The implementation of automated systems for genome editing addresses these challenges, allowing researchers to cover the increasing need and perform large-scale studies for disease modeling, drug development, and personalized medicine. In this study, we developed an automated CRISPR/Cas9-based genome editing process on the StemCellFactory platform. We implemented a 4D-Nucleofector with a 96-well shuttle device into the StemCellFactory, optimized several parameters for single cell culturing and established an automated workflow for CRISPR/Cas9-based genome editing. When validated with a variety of genetic backgrounds and target genes, the automated workflow showed genome editing efficiencies similar to manual methods, with indel rates of up to 98%. Monoclonal colony growth was achieved and monitored using the StemCellFactory-integrated CellCelector, which allowed the exclusion of colonies derived from multiple cells or growing too close to neighbouring colonies. In summary, we demonstrate the successful establishment of an automated CRISPR/Cas9-based genome editing process on the StemCellFactory platform. The development of such a standardized and scalable automated CRISPR/Cas9 system represents an exciting new tool in genome editing, enhancing our ability to address a wide range of scientific questions in disease modeling, drug development and personalized medicine.

## Introduction

The revolutionary technology of reprogramming somatic cells into human induced pluripotent stem cells (hiPSCs) offers unprecedented opportunities for disease modeling, drug development, and personalized medicine (Takahashi et al., 2007; Yu et al., 2007). HiPSCs share key features with human embryonic stem cells (ESCs), including morphology, gene expression profile, and the ability to differentiate into multiple cell lineages (Guenther et al., 2010). These specific characteristics, combined with easy accessibility of patient-specific somatic cells on demand, have made hiPSC-based cell models a preferred tool for disease modeling, drug discovery and other applications. In parallel, a substantial number of genetic variants contributing to the pathogenesis of numerous diseases have been identified. However, distinguishing between the effects of the causative mutation and the genetic background of these cells is a major challenge. To overcome this issue, RNA-guided engineered nuclease CRISPR (clustered regularly interspersed short palindromic repeats)/Cas9 (CRISPR-associated protein 9) has emerged as highly-efficient gene editing method for insertion and removal of pathogenic variants in the same genetic background, thereby enabling isogenic pair of mutant and control cells (Heidenreich and Zhang, 2016).

The increasing global use of hiPSCs and genome editing technology, coupled with the growing demand for high-quality hiPSCs and refined editing processes, has created a need for going beyond the predominant manual generation and editing of hiPSCs (Sandoe and Eggan, 2013). Despite its clear methodological structure, the gene editing process consists of multiple individual steps that are time-consuming and prone to errors. Moreover, the growing demand for gene editing in many fields is bound to necessitate an ever-growing number of trained scientific staff in order to generate large numbers of genetically modified cell lines. However, even with trained scientific staff, inter-individual differences are hardly compensable. Thus, there is a need for standardized and automated production and genome editing of hiPSCs.

Nowadays, modular robotic systems have been developed to automate the reprogramming, expansion and differentiation of hiPSC lines, enabling large-scale studies with minimized biological and technical variability (Kami et al., 2013; Konagaya et al., 2015; Paull et al., 2015; Archibald et al., 2016; Crombie et al., 2017; Kikuchi et al., 2018; Elanzew et al., 2020; Tristan et al., 2021). In recent years, we have developed the StemCellFactory, a modular system for standardized and automated reprogramming and expansion of hiPSCs (Elanzew et al., 2020; Piotrowski et al., 2021). This system comprises diverse modules connected by a robotic arm for handling different cell types in multititer plates (MTP). All devices are integrated into the COPE (Control, Optimize, Plan, Execute) software system, which controls the entire processes, including data tracking, computation of metrological data, prospective consumable management, and two-stage error handling (Jung et al., 2018b). With the establishment of a trained deep learning algorithm, unbiased detection of dead or differentiated cells, as well as the identification of other cell culture parameters, such as cell-free areas and hiPSC colony size, is feasible (Piotrowski et al., 2021). This enables an automated, user-independent confluence-based splitting procedure to account for clone-dependent differences in growth rate.

In this study, we present the development of an automated approach for CRISPR/Cas9-based genome editing of hiPSCs using the StemCellFactory. To achieve this, a 4D-Nucleofector together with a 96-well shuttle device (Lonza) were implemented into the StemCellFactory platform, and the protocols required for genome editing were adapted to the consumables and capabilities of the StemCellFactory. A flowchart outlining the steps of the automated process was created, and several parameters for single cell culturing were optimized. Finally, the effectiveness of the automated protocol was successfully validated using different cell lines and editing targets.

## Material and methods

### Human induced pluripotent stem cell culture

HiPSC lines were cultured in StemMACS iPS-Brew XF medium (iPS-Brew; Miltenyi Biotec, Bergisch Gladbach, Germany) on Geltrex (180 μg/mL; Thermo Fisher Scientific, Waltham, Massachusetts) or Matrigel (following manufacturer´s instructions, Corning, New York) coated 6-well tissue culture plates (Nunc, Thermo Fisher Scientific) with daily medium changes and regular passaging using EDTA (0.05 mM; Sigma Aldrich, St. Louis, Missouri)/PBS (Gibco, Thermo Fisher Scientific) or Accutase (1 mg/mL, Thermo Fisher Scientific). Cultures were tested for *mycoplasma* contamination and were maintained *mycoplasma* free. All hiPSC lines used in this study are certified by hPSCreg (Supplementary Table S1).

### Preparation of ribonucleoprotein complexes and single cell solution

CRISPR/Cas9 genome editing experiments were conducted using target gene-specific CRISPR-RNA (crRNA, IDT, Coralville, Iowa) (Supplementary Table S2) and trans-activating crRNA (tracrRNA, IDT). For nucleofection, an optimized protocol based on the P3 Primary Cell 96-well Nucleofector Kit (Lonza, Basel, Switzerland) was used. In brief, the guide RNA (gRNA) complex was prepared by heating crRNA and tracrRNA (200 µM each) in a 1:1 ratio at 95°C for 5 min, followed by cooling to room temperature (RT) for 15 min. Subsequently, the active CRISPR/Cas9 complex (RNP complex) was formed by incubating gRNA (100 µM) and HiFi Cas9 Nuclease V3 (IDT) in a 3:2 ratio for 45 min at RT. The P3 nucleofection buffer (Lonza) was prepared and mixed with the electroporation enhancer (IDT) according to the kit’s instructions. To obtain single cells, hiPSCs were dissociated using Accutase and manually counted.

### Manual CRISPR/Cas9 genome editing

For manual nucleofection, hiPSCs were incubated in iPS-Brew medium supplemented with ROCK inhibitor Y-27632 (RI, 10 μM; Hiss Diagnostics, Freiburg, Germany) for 1 hour. Afterwards, cells were washed once with PBS and harvested using Accutase (1 mg/mL) for 10 min at 37°C. Cells were then resuspended in an appropriate volume of PBS and pelleted by centrifugation for 3 min at 300 g. Subsequently, the cells were counted using a Neubauer counting chamber, a cell pellet with 3*10^5^ hiPSCs per condition was resuspended in 20.5 µL P3 buffer and mixed with 4 µL RNP complex. The cell suspension was transferred into a 96-well Nucleocuvette plate (Lonza) and the nucleofection was performed with a 4D-Nucleofector (Core and X Unit, Lonza) using CM150 as program code. The optimal conditions for nucleofection, considering both efficiency and viability, were tested in advance using three different electroporation programs (CA137, DN100, and CM150). HPRT-specific gRNA (IDT), known for producing high indel rates, served as a positive control. Cell viability was assessed using Trypan blue staining and a Neubauer counting chamber. After nucleofection, the cell suspension was directly mixed with 100 µL iPS-Brew medium supplemented with RI Y-27632 (10 µM). For culturing monoclonal single cell colonies, 2 μL cell suspension was seeded into a 6-well tissue culture plate and for the polyclonal nucleofection controls, 40 μL cell suspension was transferred into a 24-well tissue culture plate. Both plates were previously coated with Laminin 521 (10 μg/mL; BioLamina, Sundbyberg, Sweden) and prefilled with 2 mL and 0.5 mL iPS-Brew medium supplemented with CloneR (1x; STEMCELL Technologies, Vancouver, Canada), respectively. These optimized conditions for cultivating single cells were evaluated through preliminary experiments. Different growth matrices (Matrigel and Laminin 521) and cytoprotective media additives (CloneR and Y27632) were tested for their ability to support hiPSC survival. Briefly, hiPSC colonies were dissociated into single-cell suspensions using Accutase. The cells were seeded at densities of 100, 500, or 1,000 cells per well in 6-well tissue culture plates with varying coatings and media supplements (Matrigel ± CloneR and Y27632; Laminin 521 ± CloneR and Y27632). The medium was refreshed daily with fully supplemented iPS-Brew medium. After 4 days, the number of adherent single cells or colonies was assessed by microscopic inspection using an EVOS FL microscope (Advanced Microscopy Group, AMG).

### Automated CRISPR/Cas9 genome editing

The automated nucleofection on the StemCellFactory was based on the manual protocol with adjusted steps and volumes. The protocol was divided into three separated liquid handling subprocesses associated with specific material transport steps: (1) cell preparation, (2) nucleofection and (3) cell seeding followed by automated expansion as described in (Elanzew et al., 2020). In advance, a 50 mL Falcon tube with 4.5*10^5^ cells/mL in iPS-Brew medium supplemented with RI Y-27632 (10 µM) per approach (cells were harvested following the identical procedures described in the manual section), a 50 mL Falcon tube with iPS-Brew medium supplemented with RI Y-27632 (10 µM), a 96-deep well plate (Nunc), a 96-well Nucleocuvette plate (Lonza), a 96-V-bottom plate (Nunc) including the RNP complex (5 µL per condition), a Nunc 96-V-bottom plate including the P3 Buffer (40 µL per condition) as well as precoated 6-well and 24-well tissue culture plates were prepared and placed on the disposable hotel of the StemCellFactory.

For starting the automated CRISPR/Cas9 genome editing, the 50 mL Falcon tube with cell suspension and the 96-deep well plate were automatically transferred to the liquid handling unit (LHU) via the robotic arm and the cell preparation (1) was started. The cell suspension was resuspended and 1 mL cell solution (4.5*10^5^ cells) was pipetted into one well of the 96-deep well plate per condition. The 96-deep well plate was transferred to the centrifuge following by a centrifugation step at 300 g for 5 min. Meanwhile, additional material (the 50 mL Falcon tube with iPS-Brew medium, the 96-well Nucleocuvette plate, two prepared 96-V-bottom plates with RNP complex solution and P3 buffer, respectively) were transported to the LHU. After replacing the 96-deep well plate back onto the LHU, the liquid handling method nucleofection (2) was started. The supernatant of the cell pellet was removed, the cells were resuspended in 30 µL P3 buffer and 20 µL of this cell suspension (3*10^5^ cells) was placed into the 96-well Nucleocuvette plate. Subsequently, 4 µL RNP complex was added to the cells and mixed gently. Afterwards, the electroporation of the cells was performed using the 96-well shuttle device of the 4D-Nucleofector with program code CM150. By pipetting 100 µL iPS-Brew medium supplemented with RI Y-27632 (10 µM) to the nucleofected cells, subprocess two ended. The final subprocess of the editing procedure was defined as cell seeding (3). Both 96-V-bottom plates and the 96-deep well plate were transported back to the consumable hotel and four 6-well and one 24-well tissue culture plates were placed on the LHU. The coating medium of each well was automatically replaced with iPS-Brew medium (2 mL/6-well and 0.5 mL/24-well). Afterwards, the cell suspension of nucleofected cells was gently mixed and 2 µL per condition was transferred into one single well of the 6-well plate and 40 µL per condition was filled into one single well of the 24-well plate. The entire genome editing process ended by transporting the 6-well and 24-well plates into the incubator.

### Genotyping of polyclonal hiPSCs

To identify the overall editing efficiency, genotyping of polyclonal hiPSCs was performed by Sanger sequencing. For DNA extraction and amplification, the Phire Animal Tissue Direct PCR Kit (Thermo Fisher Scientific) was used following the manufacturer’s instructions. Polyclonal hiPSCs were harvested from the 24-well tissue culture plate after 4–5 days using Accutase treatment at 37°C for 8 min. After centrifugation at 300 *g* for 5 min, the cell pellet was resuspended in 20 μL dilution buffer with 0.5 μL DNARelease additive and incubated for 5 min at RT followed by 2 min at 98°C. Afterwards, 2.5 µL of each sample was mixed with a standard PCR master mix composed 25 µL 2x Phire animal tissue PCR buffer, 2.5 µL primer mix (10 µM of each primer, Supplementary Table S3), 1 µL Phire hot start II DNA polymerase and 19 µL H_2_O to get a final volume of 50 µL. As negative control the sample was replaced with H_2_O, and as positive control a control primer mix provided in the kit was used. The PCR protocol was 98°C for 5 min followed by 40 cycles of 98°C for 5 s, a primer-specific annealing temperature (Supplementary Table S3) for 5 s, and 72°C for 30 s. A final elongation step of 1 min at 72°C was performed. To control the amplicon size, 5 µL PCR product was run on a 1% agarose gel for 50 min at 100 V. Positive candidates were then purified with the Wizard SV Gel and PCR Clean-Up System (Promega, Madison, Wisconsin) according to the manufacturer’s instruction. In brief, 45 µL PCR product was mixed with 45 µL membrane binding solution and pipetted on a SV mini column filter. After incubation, the filter membrane was washed twice with membrane wash solution and the purified DNA was collected in 25 µL nuclease-free water. For Sanger sequencing, 7.5 ng/μL (100–500 bp) or 15 ng/μL (500–1,000 bp) PCR product in a total volume of 12 µL was mixed with 3 µL of one specific primer (10 μM forward or reverse primer). The Sanger sequencing process was performed by Microsynth SEQLAB and raw sequences were analyzed to determine the indel rate, which represent the frequency of DNA insertions or deletions, using TIDE (https://tide.nki.nl/) online software.

### Automated isolation of monoclonal cell colonies

In preparation for the automated isolation of monoclonal cell colonies, the 6-well tissue culture plate was imaged on the StemCellFactory-integrated CellCelector (ALS Automated Lab Solutions, Jena, Germany) 4 hours after seeding and on each following day for 6–8 days. The images were analyzed to detect colonies which grew from a single nucleofected cell and had a minimum distance of 500 µm to neighbor colonies. The process for automated cell colony isolation was performed as described (Elanzew et al., 2020). In brief, a source 6-well tissue culture plate, a precoated target 24-well tissue culture plate filled with 0.5 mL iPS-Brew medium per well, a CellCelector tray with scrape capillaries and an empty CellCelector tray were loaded onto the CellCelector. Monoclonal cell colonies that met the defined criteria were automatically identified or manually selected by the user and a picking list was generated. For the isolation, the monoclonal cell colonies were first detached by scraping the colony with a crosswise movement using individual scrape capillaries following by aspiration into the scrape capillaries. Next, the isolated cell fragment was dispensed into the target 24-well plate. During the process, images of the colony position were acquired automatically before and after cell isolation to validate successful detachment. Afterwards, the 24-well plate was transported into the incubator and the monoclonal cells were expanded for 5–7 days with a daily medium change.

### Control software COPE

To operate the integrated hardware devices of the StemCellFactory in one single control software, we developed the software COPE (Control, Optimize, Plan, Execute; Fraunhofer IPT, Aachen, Germany), which enables the execution of individual commands, creation of production processes, data handling and visualization of collected data (Jung et al., 2018a; Jung et al., 2018b; Doulgkeroglou et al., 2020; Elanzew et al., 2020). Individual hardware-specific protocols such as open platform communication unified architectures (OPC-UA), several programmable logic controller (PLC) software development kits (SDK) or others to communicate with external programs were embedded in one system by using separate software agents for every device. The generation of this agent-based architecture allows adding a new device by changing or reprogramming a software agent without affecting the control software itself. COPE is a service-oriented software that assigns different services to the individual hardware devices and the user can combine all services into a workflow. Furthermore, measurement data and consumables data are permanently saved in a specific Structured Query Language (SQL) database. For the automated genome editing process on the StemCellFactory the 96-well shuttle device was integrated into COPE via a new software agent. In addition to the standardized commands “initialize” and “error quit”, the nucleofection can be started via “start_process” with the optimized, predefined program settings.

## Results

In this study, we aimed at establishing an automated CRISPR/Cas9-based genome editing process on the StemCellFactory by following a three-step strategy: Initially, we determined the required technical equipment for an automated process and implemented an automatable nucleofector, a specific transport holder for MTPs and new pipetting tips. Subsequently, a widely used manual protocol (Bertan et al., 2021; Wen et al., 2023) for genome editing and single-cell culturing was transcribed into an automated workflow and finally, we validated the newly established, automated CRISPR/Cas9-based genome editing process in comparison to manual handling. Furthermore, optimized clone picking criteria were established to ensure the selection of monoclonal colonies.

### Integration of an automatable nucleofector and adaptation of consumables

**FIGURE 1:**
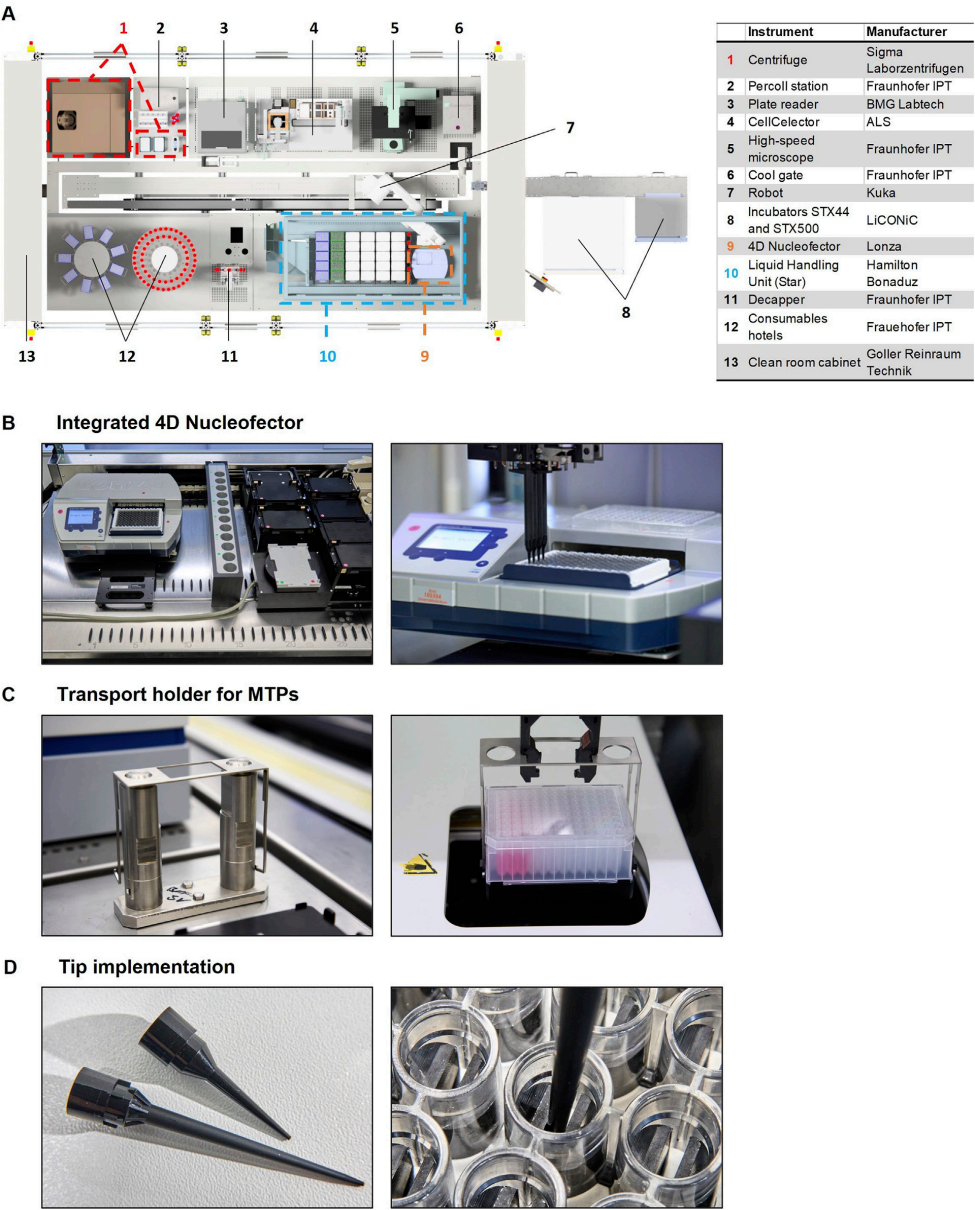
StemCellFactory setup and modifications for automated genome editing. **(A)** Left: Schematic overview of integrated modules on the StemCellFactory together with a list of instruments and manufacturers. All instruments are connected via the robotic arm and controlled by the control software COPE. Newly implemented instruments and modifications for automated genome editing are highlighted: centrifuge and transport position (red), liquid handling unit (blue) and 4D-Nucleofector (orange). **(B)** New integrated 96-well shuttle device of the nucleofector on the liquid handling unit, **(C)** the inhouse manufactured specific holder for the transport of 96-DW plates and **(D)** new implemented 10 μL and 50 µL pipette tips characterized by an elongated, slender shape, enabling precise handling of small volumes (as low as 1 µL) and accurate placement and mixing of liquids in the center of each well of the 96-well Nucleocuvette plate.

The previous technical set-up of the StemCellFactory for automated reprogramming and expansion of hiPSCs offers a well-established basis for developing an automated CRISPR/Cas9-based genome editing process. Relevant hardware devices including incubators, a liquid handling unit, a centrifuge, a microscope and a CellCelector had already been integrated and interconnected through a robotic arm (Figure 1A). To harness the StemCellFactory for automated genome editing, a 4D-Nucleofector in conjunction with a 96-well shuttle device (Lonza) was implemented (Figures 1A,B). The 96-well shuttle was directly mounted on the deck of the liquid handling unit to enable fast and efficient transport of required liquids, thereby minimizing process duration and maximizing cell viability. The software integration of the nucleofector was accomplished using COPE.

Several further adjustments were made to translate the manual genome editing protocol for execution on the automation platform. Manually used consumables were replaced with materials suitable for scalable and parallelizable processes, which could be handled by the installed modules. Single reaction tubes and 15-mL Falcon tubes were substituted with various 96-well MTP formats and 50-mL Falcon tubes, respectively, to allow a high number of editing reactions to be performed in parallel. Moreover, a special holder was newly fabricated to facilitate the centrifugation step of 96-deep well plates, enabling the placement and replacement of rectangular buckets together with the 96-deep well plates in the centrifuge (Figure 1C). Another modification involved implementing 10 μL and 50 µL pipetting tips (Hamilton, Bonaduz, Switzerland) characterized by an elongated, slender shape, enabling precise handling of small volumes (as low as 1 µL) and accurate placement and mixing of liquids in the center of each well of the 96-well Nucleocuvette plate, where the distance between the electrodes is minimal (Figure 1D).

### Definition of optimal conditions for automated genome editing

To establish an automated genome editing workflow, we followed a well-established manual protocol previously used by Bertan et al., 2021. However, the precise selection of distinct parameters is of paramount importance in achieving high indel rates (frequency of insertions or deletions in a DNA sequence) without compromising cell viability. Therefore, initial experiments were manually performed testing three different nucleofection programs (CA137, DN100, and CM150) on three hiPSC lines with different genetic backgrounds (1x female, 2x male, designated hiPSC1 to 3, hPSCreg names listed in Supplementary Table S1) by using an HPRT-specific gRNA that is known to result in high indel rates (positive control, IDT). Viability assessment was performed immediately after nucleofection and overall editing efficiency was assessed by quantifying indel rates of expanded polyclonal hiPSC colonies cultured on 24-well tissue culture plates. The use of the electroporation program CM150 in conjunction with P3 buffer resulted in the highest cell viability (83.5% ± 0.9%) and indel rates (81.7% ± 2.3%) (Supplementary Figure S1A, B).

In addition, the optimal conditions for the survival and growth of single hiPSCs after nucleofection were determined, as well as a favourable hiPSC seeding density for the generation of monoclonal cell lines. We compared different growth matrices (Matrigel and Laminin 521) and cytoprotective media supplements (CloneR and Y27632) for assessing their capacity to promote hiPSC survival (Supplementary Figure S1C). We identified the combination of Laminin 521 coating and iPS-Brew medium supplemented with CloneR as the best culture condition for ensuring an optimal survival of single-cell-seeded hiPSCs, resulting in a substantial number of hiPSC colonies with regular morphology after four and 10 days of expansion (Supplementary Figure S1C, D). Furthermore, we tested different seeding densities of single hiPSCs (100, 500 and 1,000 cells/well in a 6-well tissue culture plate) to determine the seeding density at which the maximum number of monoclonal colonies could be obtained. We examined the cells 5 hours after seeding and quantified the number of single cells (SC), single cells positioned too closely, which could potentially generate polyclonal hiPSC colonies (SC-C) and cell aggregates (CA) (Supplementary Figure S1E). Seeding densities of 500 and 1,000 cells/well resulted in 84,6% ± 3.6% and 80,2% ± 2.2% of isolated single cells, respectively. However, the seeding of 1,000 cells/well led to the formation of more than 450 colonies, which could lead to a reduced number of monoclonal colonies. Overall, our manually collected data showed that the CM150 electroporation program provides superior indel rates, while the laminin 521 coating together with the CloneR-supplemented iPS-Brew medium ensures optimal hiPSC survival. Furthermore, seeding 500 hiPSCs/well in a 6-well tissue culture plate guarantees the generation of monoclonal hiPSC colonies (Supplementary Figures S1A, C, E).

### Implementation of the automated workflow

**FIGURE 2:**
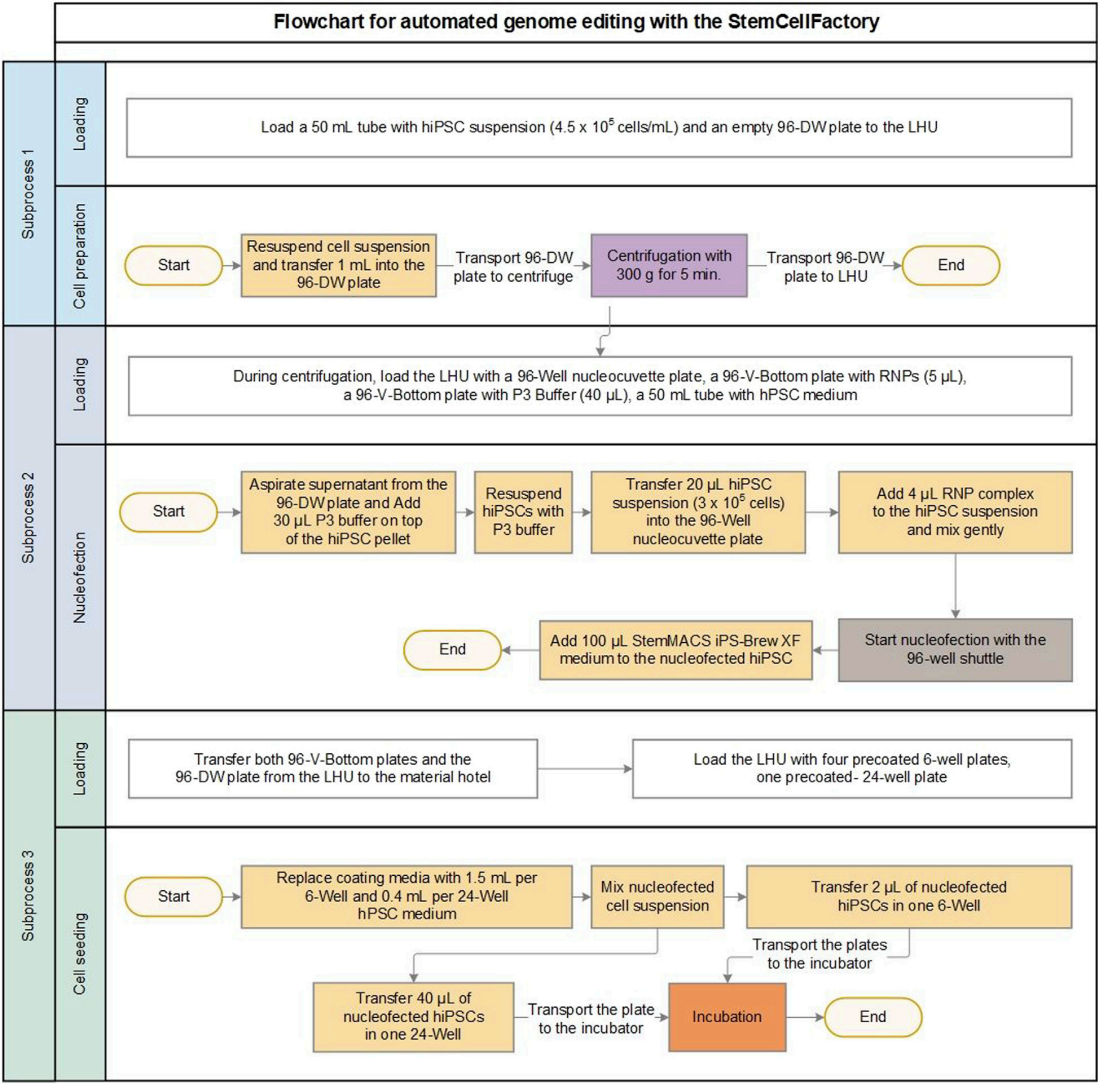
Process development for automated genome editing. Flowchart for automated genome editing allowing the user to edit of up to 24 independent conditions (e.g., 24 different target genes in 1 cell line or six target genes in 4 cell lines) in parallel using the StemCellFactory. The whole process is divided into three sub-processes (cell preparation, nucleofection and cell seeding) with associated transport steps of the required materials. In sub-process three the nucleofected cells are seeded into a 6-well and a 24-well plate. The 24-well plate serves as a polyclonal control for quantifying overall editing efficiency, while the 6-well plate is used to culture monoclonal cell colonies. Volumes are given for one editing process. Box color represents different modules: white = robot, yellow = liquid handling unit, purple = centrifuge, brown = nucleofector, orange = incubator.

Based on these conditions the manual genome editing protocol was successfully translated into an automated workflow (Figure 2). The entire automated process was divided into three subprocesses based on the involved StemCellFactory modules, consumables and the corresponding material transport steps. The deck layouts, which provide a schematic representation of the arrangement of the consumables and modules on the LHU for each subprocess, are shown in Supplementary Figure S2. The first subprocess, termed cell preparation, involved automated loading of the desired number of manually prepared singularized hiPSCs into individual wells of a 96-deep well plate using the LHU, followed by centrifugation (Supplementary Figure S2A). For the transport of the 96-deep well plate into the centrifuge, the newly manufactured holder was used (Figure 1C). Subsequently, the centrifuged 96-deep well plate, along with further required materials, such as a 96-well Nucleocuvette plate, two 96-V-bottom plates filled with RNPs and P3 buffer, and a 50 mL Falcon tube with iPS-Brew medium, were placed on the LHU, leading in the second subprocess termed nucleofection (Figure 2; Supplementary Figure S2B).

For this automated part, we meticulously tested and optimized several critical liquid handling procedures. This included the aspiration parameters of the LHU to remove as much supernatant as possible from the 96-deep well plate without losing the cell pellet. Furthermore, the volumes of RNPs and P3 buffer in the 96-V-bottom plates were carefully assessed to strike a balance between higher volumes, which are cost-intensive due to increased dead volumes, and small volumes, which are prone to pipetting errors due to liquid evaporation in the laminar flow system. Ultimately, we determined that providing 5 µL RNP and 40 µL P3 buffer per condition in individual wells of the 96-V-bottom plates, followed by the LHU automatically transferring 4 µL of RNP and 30 µL of P3 buffer into the wells of interest, yielded optimal results. Additionally, the newly implemented elongated, low volume pipette tips enabled enhanced precision when handling small volumes and ensured accurate positioning and thorough mixing of liquids within the central region of each well in the 96-well Nucleocuvette plate.

The final subprocess covers cell seeding of the nucleofected cells (Figure 2; Supplementary Figure S2C). After electroporation in the 4D-Nucleofector, cells were seeded onto pre-coated 6-well and 24-well tissue culture plates. For each condition, one individual well of a 6-well and a 24-well plate is required. Identical to the manual procedure, the 24-well plate serves as a polyclonal control for quantifying overall editing efficiency, while the 6-well plate was used to culture monoclonal cell colonies. The optimized single-cell seeding number of 500 cells/well were adopted from the manually performed preliminary tests (Supplementary Figure S1C). Finally, the plates were automatically transported to the incubator for the expansion of the edited clones.

The current design of the LHU used in the StemCellFactory enables placement and handling of four 6-well plates and one 24-well plate in parallel, allowing the user to edit automatically up to 24 independent conditions (e.g., 24 different target genes in 1 cell line or up to six target genes in 4 cell lines) during a single run of the entire workflow.

### Validation of the automated CRISPR/Cas9-based genome editing process

After transferring the manual protocol for CRISPR/Cas9-based genome editing into an automated process, the newly implemented automated protocol underwent biologically validation using the three manually employed hiPSC lines and various target genes. The selection of target genes involved choosing gRNAs that had been manually employed in-house and exhibited a broad spectrum of editing efficiencies, ranging from low (PLCG2) to medium (ASPA) to high indel rates (PSEN2, SYNGAP1 and NDUFS4). Following the automated genome editing process applied to the five target genes as described in the preceding section, the resulting cell colonies were expanded, harvested and manually processed to examine the indel rates.

**FIGURE 3:**
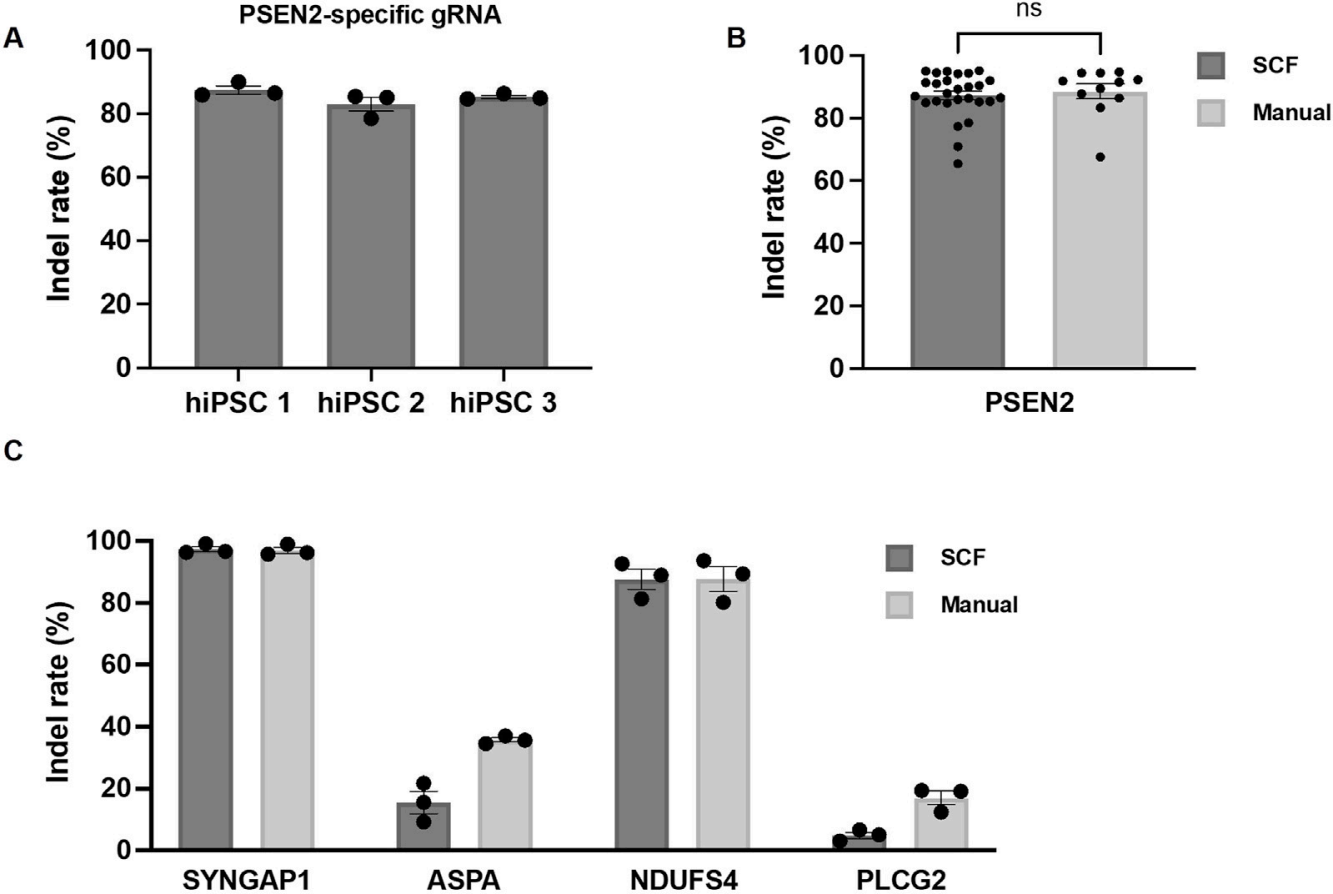
Automated genome editing using the StemCellFactory compared to manual handling. **(A)** Indel rates using a PSEN2-specific gRNA in three independent hiPSC lines (hiPSC 1, hiPSC 2, hiPSC 3) utilizing the newly implemented process on the StemCellFactory (n = 3). **(B)** Indel rates with PSEN2-specific gRNA after automated and manual processing of hiPSC 1, hiPSC 2 and hiPSC 3 (automated: n = 27; manual: n = 11). **(C)** Indel rates after automated and manual processing of hiPSC 1 using gRNAs specific for SYNGAP1, ASPA, NDUFS4 and PLCG2 (n = 3). All bar graphs data are represented as mean ± SEM.

Initially, we determined the editing efficiency after automated transfection of PSEN2 into hiPSC line one to 3. The indel rates of polyclonal colonies produced using the StemCellFactory exceeded 80% for all 3 cell lines (Figure 3A; hiPSC1 87.5% ± 1.3%, hiPSC2 83% ± 2.3%, hiPSC3 85.3% ± 0.5%). Subsequently, we compared the indel rates between automatically and manually executed experiment using PSEN2. No significant difference was detected, and the indel rates remained consistently high (Figure 3B; StemCellFactory: 87.2% ± 1.4%, manual: 88.5% ± 2.4%). Two additional gRNAs targeting SYNGAP1 and NDUFS4, which were expected to have a high editing efficiency, and gRNAs targeting PLCG2 and ASPA, which were manually judged to have a lower efficiency, were then used. As expected, the editing efficiencies varied across the four target genes, with indel rates ranging from less than 5% for PLCG2 (4.9% ± 1.0%) to over 85% for NDUFS4 (87.6% ± 3.3%) and up to 97% for SYNGAP1 (97.3% ± 0.9%) (Figure 3C). These results were consistent with the outcomes observed during manual editing and were dependent on the target gene. The transfection of ASPA into hiPSC one to three was also successful using the automated process (indel rate 15.5% ± 3.6%); however, the editing efficiency was lower compared to the manual approach (Figure 3C). Representative bar graphs of the percentage distribution of indels in the DNA sequences including chromatograms for hiPSC line 1 after automated transfection of SYNGAP1- and NDUFS4-specific RNPs are shown in Supplementary Figure S3.

### Derivation of clonal lines

**FIGURE 4:**
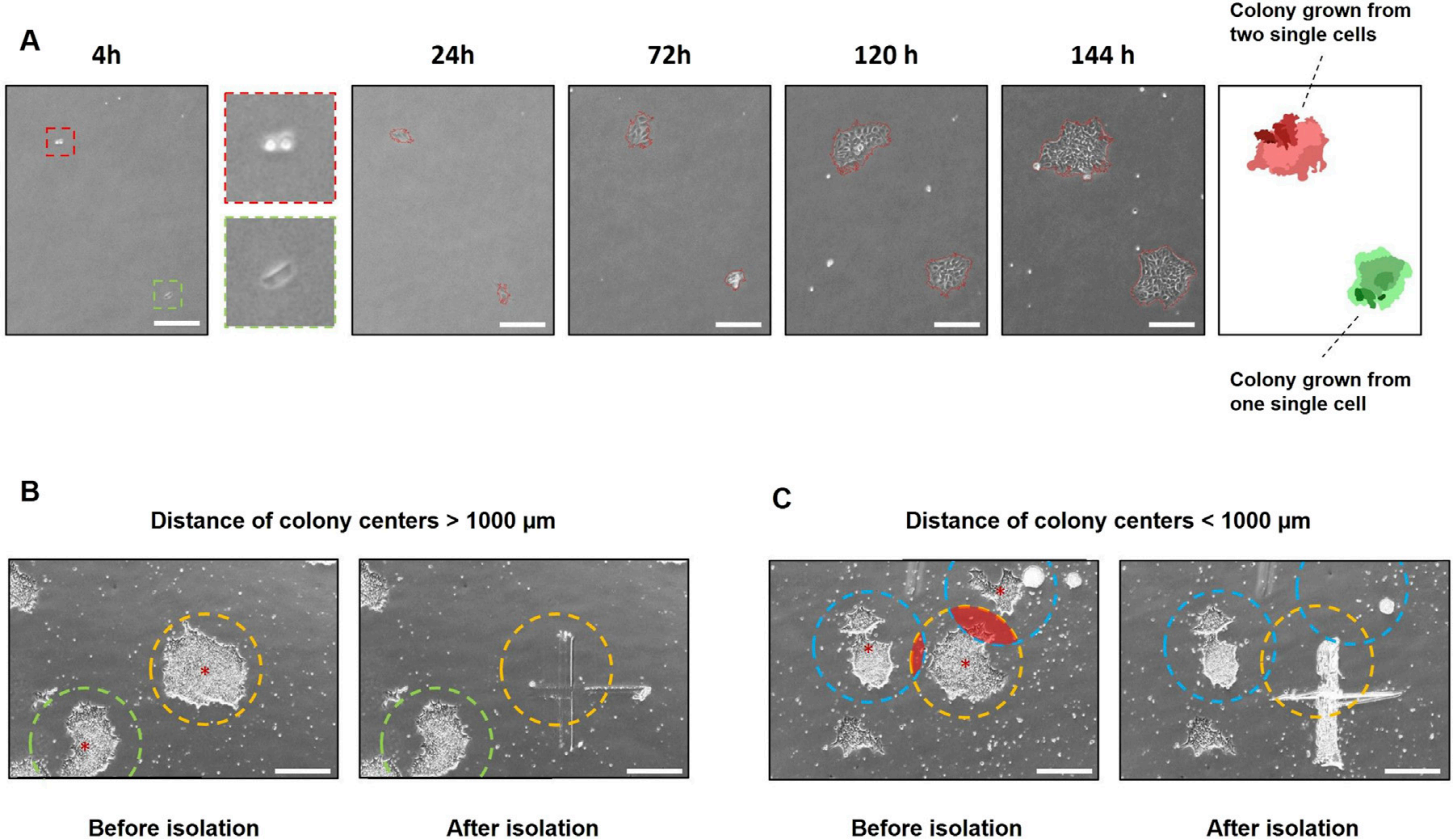
Automated tracing and isolation of monoclonal colonies using the CellCelector. **(A)** Phase contrast images of hiPSC colonies growing from single cells expanded for 6 days. The software virtually reconstructs an overlay to determine if the colonies are grown monoclonal. Green = monoclonal colony; red = polyclonal colony; scale bars = 500 µm **(B)** Colony isolation using the CellCelector. A distance >1,000 µm from the center of each colony (red stars) to the neighboring colony was chosen to enable scraping of individual monoclonal colonies. The target colony (yellow circle) was selectively scraped without contacting the neighboring colony (green circle). **(C)** Target colony (yellow circle) was scraped along with neighboring colonies (blue circle) that had grown too close to the target colony. Radius of circles = 500 μm; scale bars = 500 µm **(B, C)**.

In addition to the use of polyclonal cell lines, the cultivation of monoclonal colonies is crucial for addressing scientific questions. Therefore, the growth of colonies originating from single cells after transfection was monitored using the StemCellFactory-integrated CellCelector. A 6-well tissue culture plate containing 500 single cells was imaged on the CellCelector 4 h after seeding and subsequently every 24 h for six to 8 days, starting from the following day (Figure 4A). The internal software generated a virtual overlay of all images, enabling the identification of monoclonal colonies that grew from a single cell. Consequently, polyclonal colonies arising from more than one single cell were excluded. Another criterium for ensuring monoclonal colonies is the distance between the colonies. A distance longer than 1,000 µm from the center of each colony to its neighboring colony was selected to ensure scraping of individual monoclonal colonies (Figure 4B). If the colonies grew closer to each other, the detachment of a single colony could not be guaranteed (Figure 4C). Colonies that met the predetermined criteria were automatically detached by scraping and transferred into a coated 24-well tissue culture plate for further expansion.

Overall, these data demonstrate successful implementation of an automated CRISPR/Cas9-based genome editing process on the StemCellFactory platform. The editing efficiency, evaluated across various hiPSC lines and multiple target genes, was comparable to that of manual procedures. Furthermore, the CellCelector system enabled safeguarding of monoclonal growth and harvesting.

## Discussion

The present study aimed to establish an automated CRISPR/Cas9-based genome editing process on the StemCellFactory to address the growing demand for scalable and standardized protocols for genome-edited cells. To achieve this goal, we tackled three main challenges: identifying required technical equipment, adapting a widely used manual protocol for genome editing and monoclonal cell expansion into automated procedures, and validating the newly established process compared to manual handling.

In terms of hardware, an automatable 4D-Nucleofector in conjunction with a 96-well shuttle device was successfully integrated into the StemCellFactory. Additionally, relevant modifications have been made including the utilization of 10 μL and 50 µL pipetting tips characterized by an elongated, slender design, as well as the development of a deep well plate adapter for centrifugation steps. From the software perspective, the integration of the nucleofector was executed within the pre-existing control software COPE (Biermann et al., 2021). With these technical modifications, we were able to adapt the manual genome-editing protocol into an automated process using the StemCellFactory. While our automated process demonstrated comparable editing efficiency to manual procedures across different hiPSC lines and target genes, slight disparities were observed. Two target genes (ASPA, PLCG2) showed higher efficiency with manual handling, potentially due to recognized limitations of liquid handlers in the lower μL range and gradual reagent evaporation. Advancements in automated liquid handling techniques, e.g., specific pipetting heads for low volumes in µL and nL, may address these issues.

In addition to achieving a standardized and scalable automated process with high editing efficiency, the automated cultivation of monoclonal colonies derived from single cells is of significant importance. Mostly up to 96 individual clones of edited cells are simultaneously cultivated because researchers are uncertain about the editing efficiency but aim to produce monoclonal cell lines as fast as possible. This process is highly time-intensive and prone to errors, as each colony must be manually picked and cultured without cross-contamination. To streamline this process, we adapted these manual procedures into an automated workflow. Singularized cells were seeded at low density into 6-well plates and monitored over several hours and days using the microscope on the CellCelector. This optical verification provides a high level of confidence in identifying colonies of single cell origin. Cell colonies meeting predetermined criteria were automatically detached by scraping and transferred into fresh tissue culture plates for further expansion and analysis. With the potential cultivation of up to 96 individual colonies, any minor discrepancy in editing efficiency between manual and automated protocols becomes negligible. In the future, the integration of specific single-cell dispensing devices into the StemCellFactory could further facilitate the subcloning process. In addition, as an extra layer of safety, *post hoc* NGS-based amplicon sequencing could be used to further verify the single cell origin of the resulting clones.

While not covered by this manuscript, our setup should also be highly suitable for the introduction of, e.g., specific point mutations or larger tags via single- or double-stranded homology-directed repair (HDR) as the required templates could be easily co-nucleofected with the RNPs. However, since HDR is less efficient than generating indels, a larger number of candidate clones would have to be screened.

In terms of costs, the primary reduction occurs in individual labor efforts, as the consumables used remain consistent with those of manual procedures. However, manual activities such as clone selection and picking are time-consuming that automation results in significant cost benefits. Especially when considering the scalability of the process to meet the increasing demand for genome edited cells in the future, there is often a lack of standardization and reproducibility, while simultaneously increasing the risk of human errors.

Laboratory automation has gained widespread acceptance in recent years and is a gold standard in many laboratories. Often the automation of molecular biological processes in genomics or diagnostics is still in the focus, but interest in automated cell culture handling is steadily increasing. The StemCellFactory continues to represent a particularly appealing automated cell culture system in terms of its diversity and complexity, which is currently not comparable with other systems (Elanzew et al., 2020). With a wide range of technical equipment (consumable hotel, incubators, centrifuge, clone picker, liquid handler, high-content microscope, etc.), a large number of cell culture processes can be automated. Starting with the reprogramming of iPSCs, extending to monoclonal cell picking and expansion including quality control with artificial intelligence, cell editing has become attainable on the StemCellFactory. The combination of these crucial cell culture processes is a unique selling point of the StemCellFactory.

Currently, the editing capacity is restricted to 24 edits in parallel due to spatial constraints in the deck layout. By adapting the deck layout and optimizing the individual pipetting steps, it became feasible to use the full capacity of a 96-well nucleofection plate, enabling parallel processing of 96 edits together with an increase in overall efficiency. In order to achieve robust processing of up to 96 individual cell lines with up to 96 genes, the process must be further optimized. This could help many institutes and pharma companies regarding personalized medicine.

Taken together, we automated CRISPR/Cas9-based genome editing on the StemCellFactory to meet the rising demand for scalable protocols. Challenges included equipment identification, protocol adaptation, and validation against manual handling. Integration of a 4D-Nucleofector directly on the liquid handling system and protocol optimizations enabled automation. While efficiencies were comparable, disparities in editing of certain genes highlighted technical limitations. Nevertheless, automation of genome editing and monoclonal colony cultivation streamlined the process, reducing errors and time. With potential cost savings and increased reproducibility, further optimizing the process could benefit personalized medicine efforts in various institutions and pharmaceutical companies.

## Data Availability

The datasets presented in this study can be found in online repositories. The names of the repository/repositories and accession number(s) can be found in the article/Supplementary Material.

## Supplementary material

Supplementary Table S1 List of used cell lines.

Supplementary Table S2 List of used CRISPR-Cas9 crRNA.

Supplementary Table S3 List of used primer pairs including PCR product size and annealing temperature.

Supplementary Figure S1 Evaluation of nucleofection parameters and modification of coatings and medium to improve single cell survival. **(A)** Indel rates with HPRT-specific gRNA and **(B)** viability of three hiPSC lines with different genetic backgrounds (1x female, 2x male) after using three different nucleofection programs (CA137, DN100, CM150) (A, n = 3; B, n = 2). **(C)** Number of colonies formed 4 days after seeding of single hiPSCs. hiPSCs were seeded at a density of 100, 500 or 1,000 cells/well in a 6-well tissue culture plate with different coatings (Matrigel, Laminin 521) and cytoprotective media supplements (Y-27632, CloneR). **(D)** Example colonies with pluripotent characteristics after four and 10 days of expansion in CloneR-supplemented culture media on a 6-well tissue culture plate coated with Laminin 521. Scale bars = 500 µm **(E)** Percentage of adherent cells 5 hours after single hiPSC seeding. The number of single cells (SC), single cells positioned too closely, which could potentially generate polyclonal hiPSC colonies (SC-C) and cell aggregates (CA) was quantified (n = 4). Scale bars = 100 µm. All bar graphs data are represented as mean ± SEM.

Supplementary Figure S2 Deck layout of the Liquid Handling Unit during automated genome editing. **(A)** Deck layout of sub-process 1 (cell preparation) with the cell suspension in a 50 mL Falcon tube (red) and a 96-deep well plate (blue) with lid (yellow). **(B)** Deck layout of sub-process 2 (nucleofection) with a 96-deep well plate (blue) containing centrifuged cell pellets, two 96-V-bottom plates (green) containing RNPs and P3 buffer, a Nucleocuvette™ plate (purple) and a 50 mL Falcon tube (red) containing medium. Lids are highlighted in yellow. **(C)** Deck layout with four coated 6-well plates (green), one coated 24-well plate (orange), a Nucleocuvette™ plate (purple) with nucleofected cells and a 50 mL Falcon tube (red) containing medium. Lids are highlighted in yellow.

Supplementary Figure S3 Representative analysis of Sanger sequencing results. Quantification of editing efficiency for **(A)** SYNGAP1 and **(B)** NDUFS4 after automated transfection of RNPs into hiPSC line 1. The bar charts show the percentage distribution of insertions and deletions in the DNA sequences of polyclonal lines. The chromatograms display the edited and wild-type (control) sequences in the region around the specific guide sequence. The horizontal black underlined region represents the guide sequence. The horizontal red dotted underline marks the location of the PAM site. The vertical black dotted line represents the predicted cut site. The bar charts are created using TIDE (https://tide.nki.nl/) and the chromatograms using ICE (https://ice.synthego.com/#/).
