# Supplementary figures and images for: Automated CRISPR/Cas9-based genome editing of human pluripotent stem cells using the StemCellFactory
Source: Front Bioeng Biotechnol. 2024 Sep 20;12:1459273. doi: 10.3389/fbioe.2024.1459273 (PMC11449837; doi:10.3389/fbioe.2024.1459273)

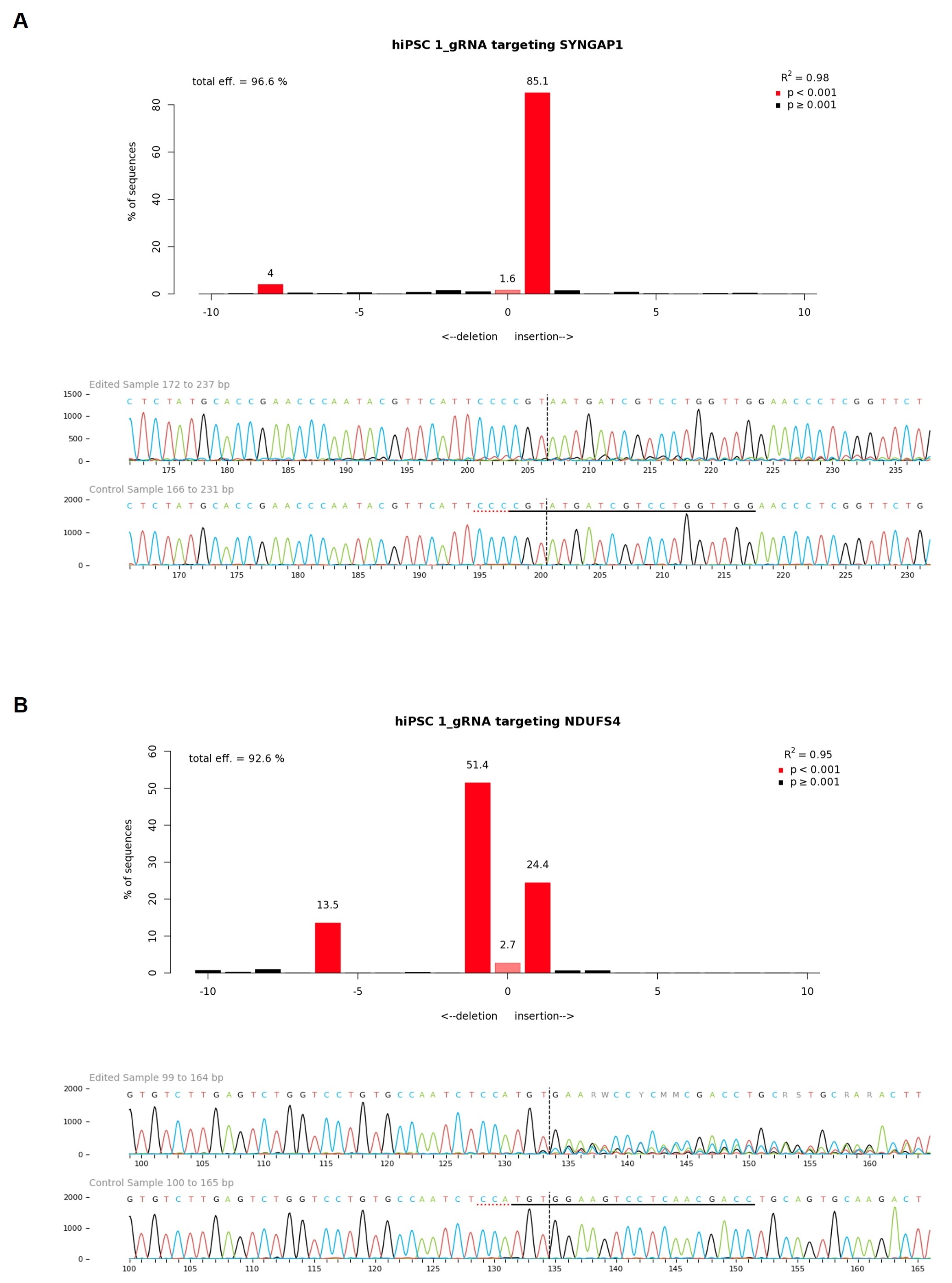

Supplement: Supplementary file 1 [file Image3.JPEG]

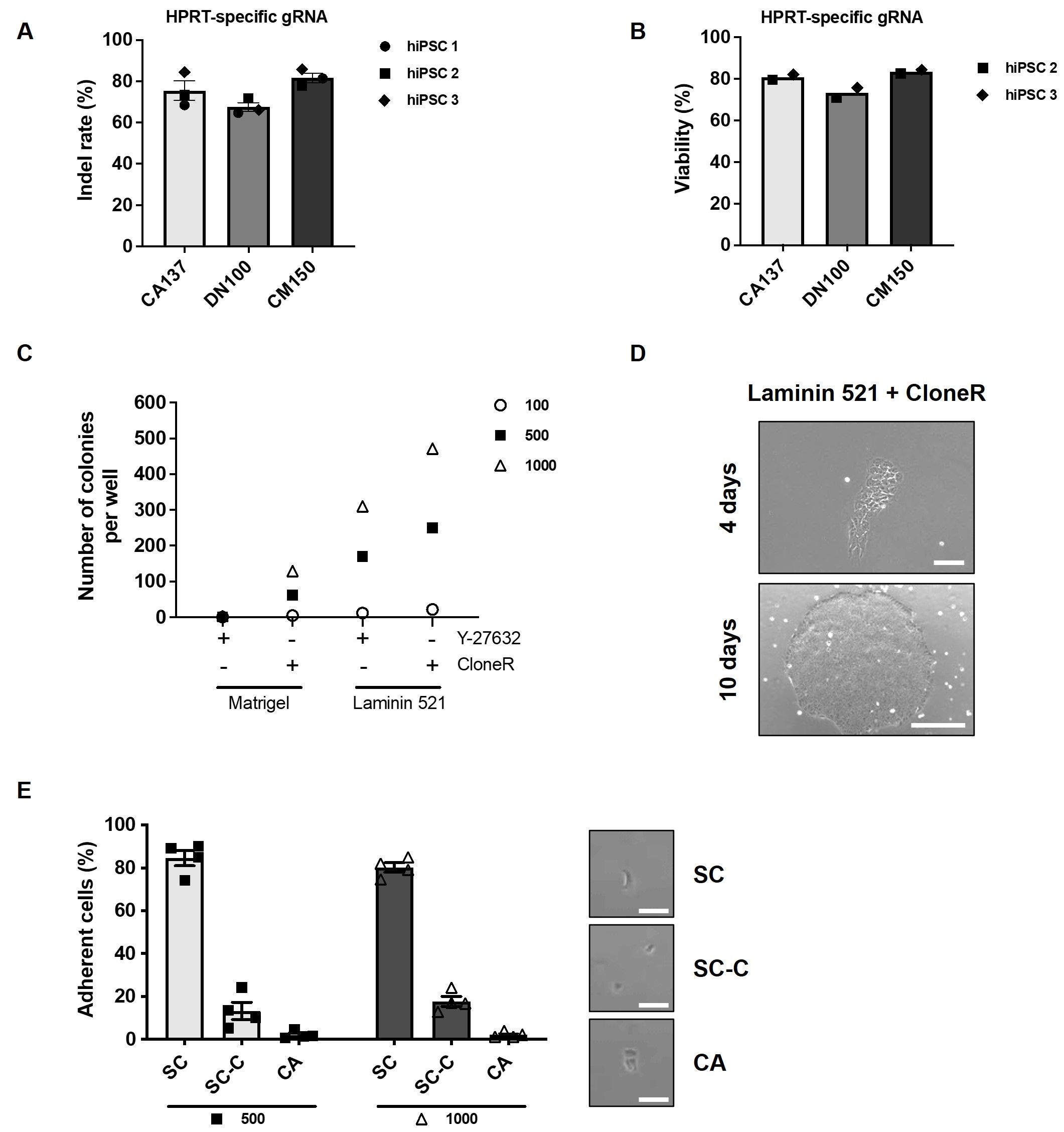

Supplement: Supplementary file 4 [file Image1.JPEG]

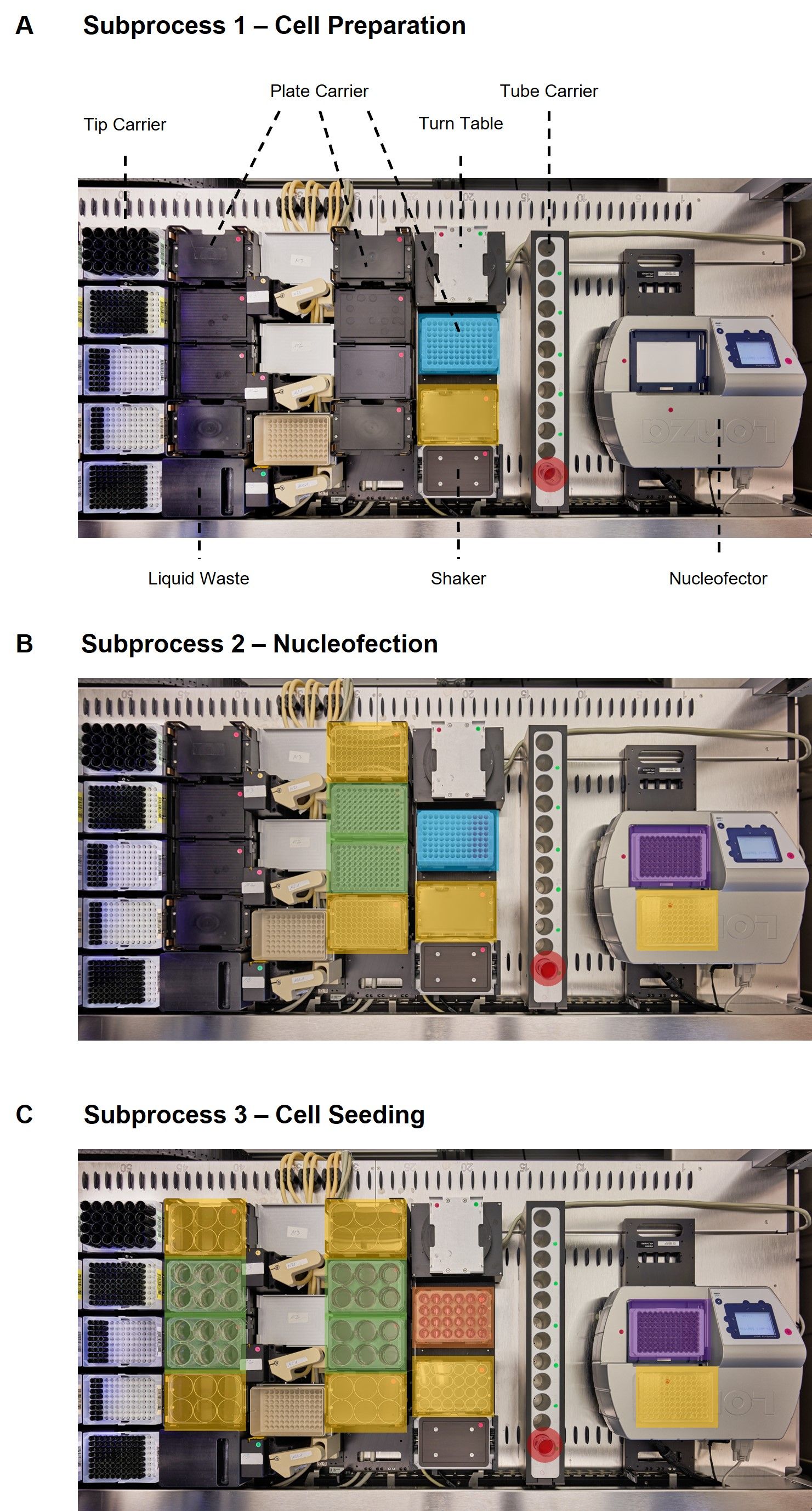

Supplement: Supplementary file 5 [file Image2.JPEG]
