# Supplementary material for: Automated CRISPR/Cas9-based genome editing of human pluripotent stem cells using the StemCellFactory
Source: Front Bioeng Biotechnol. 2024 Sep 20;12:1459273. doi: 10.3389/fbioe.2024.1459273 (PMC11449837; doi:10.3389/fbioe.2024.1459273)
Supplement: Supplementary file 2 [file Table1.pdf]

Supplementary Table 1 : List of used cell lines

Cell line	hPSCreg name and link	Internal name	Generator
hiPSC 1	UKBi005-A	iLB-C-31f-r1	University Hospital of Bonn
hiPSC 2	UKBi006-A	iLB-C-35m-r1	University Hospital of Bonn
hiPSC 3	UKBi013-A	iLB-C-133bm-s4	University Hospital of Bonn
hiPSC 4	UKBi017-A	iLB-C-14m-s11	University Hospital of Bonn
