# Supplementary material for: Automated CRISPR/Cas9-based genome editing of human pluripotent stem cells using the StemCellFactory
Source: Front Bioeng Biotechnol. 2024 Sep 20;12:1459273. doi: 10.3389/fbioe.2024.1459273 (PMC11449837; doi:10.3389/fbioe.2024.1459273)
Supplement: Supplementary file 3 [file Table2.pdf]

Supplementary Table 2: List of used CRISPR-Cas9 crRNA

**Target gene**	**Sequence**
PSEN2	CAT GAT GAG GGT GTT CAG CA
SYNGAP1	CCA ACC AGG ACG ATC ATA CG
ASPA	AAG AGT GCA CCC CAT GTT AG
NDUFS4	GGT CGT TGA GGA CTT CCA CA
PLCG2	GTT CTG TAG GGG GTA TAT CC
