# Supplementary material for: Automated CRISPR/Cas9-based genome editing of human pluripotent stem cells using the StemCellFactory
Source: Front Bioeng Biotechnol. 2024 Sep 20;12:1459273. doi: 10.3389/fbioe.2024.1459273 (PMC11449837; doi:10.3389/fbioe.2024.1459273)
Supplement: Supplementary file 6 [file Table3.pdf]

Supplementary Table 3: List of used primer pairs including PCR product size and annealing temperature

Primer	Sequence	Size of PCR product [bp]	Annealing temperature [°C]
ASPA-fwd	TGG CGA CTG GTT CTT TTT ACA C	400	62
ASPA-rev	TCT CCT GCA CCT TCC CTC AT		
NDUFS4-fwd	GCA GCC TGT TGT GAA CTG TC	401	64
NDUFS4-rev	TCA AAT CAC TTT CCT GAA AAC CTG G		
PLCG2-fwd	CCA TAA ATG AGG GCT CTC AGG AGT	438	64
PLCG2-rev	CCT CAT CGG TTG GCA TGG AGA		
PSEN2-fwd	GGT GCC AGG AAA TGA GCT GG	862	65
PSEN2-rev	CCA ATG AGG AAC TCA AGT GG		
SYNGAP1-fwd	CCC CTT TGT ATA AAT GCT TGT CTT	400	62
SYNGAP1-rev	TGC AAA TGT TTC TTC TTT GTC TGC		
